# From whole-organ imaging to in-silico blood flow modeling: A new multi-scale network analysis for revisiting tissue functional anatomy

**DOI:** 10.1371/journal.pcbi.1007322

**Published:** 2020-02-14

**Authors:** Pol Kennel, Jules Dichamp, Corinne Barreau, Christophe Guissard, Lise Teyssedre, Jacques Rouquette, Julien Colombelli, Anne Lorsignol, Louis Casteilla, Franck Plouraboué

**Affiliations:** 1 Institute of Fluid Mechanics of Toulouse (IMFT), Toulouse University, CNRS, INPT, UPS, Toulouse, France; 2 CNRS 5273; UMR STROMALab, BP 84225, F-31 432 Toulouse Cedex 4, France; 3 ITAV, Université de Toulouse, CNRS, UPS, France; 4 Advanced Digital Microscopy Core Facility, Institute for Research in Biomedicine (IRB Barcelona), The Barcelona Institute of Science and Technology. C. Baldiri Reixac, 10. E-08028 Barcelona, Spain; University of California San Diego, UNITED STATES

## Abstract

We present a multi-disciplinary image-based blood flow perfusion modeling of a whole organ vascular network for analyzing both its structural and functional properties. We show how the use of Light-Sheet Fluorescence Microscopy (LSFM) permits whole-organ micro-vascular imaging, analysis and modelling. By using adapted image post-treatment workflow, we could segment, vectorize and reconstruct the entire micro-vascular network composed of 1.7 million vessels, from the tissue-scale, inside a ∼ 25 × 5 × 1 = 125mm^3^ volume of the mouse fat pad, hundreds of times larger than previous studies, down to the cellular scale at micron resolution, with the entire blood perfusion modeled. Adapted network analysis revealed the structural and functional organization of meso-scale tissue as strongly connected communities of vessels. These communities share a distinct heterogeneous core region and a more homogeneous peripheral region, consistently with known biological functions of fat tissue. Graph clustering analysis also revealed two distinct robust meso-scale typical sizes (from 10 to several hundred times the cellular size), revealing, for the first time, strongly connected functional vascular communities. These community networks support heterogeneous micro-environments. This work provides the proof of concept that in-silico all-tissue perfusion modeling can reveal new structural and functional exchanges between micro-regions in tissues, found from community clusters in the vascular graph.

## Author summary

New optical microscopy called light-sheet fluorescence microscopy (LSFM) allows for 3D optical scan imaging of an entire tissue, at the micron scale, for reconstructing its complete microvascular bed. The 3D reconstruction of a vectorized vascular network can display as many as 1.7 million vessels inside a 125mm^3^ volume of mouse fat tissue. This 3D reconstruction allows for vessel visualisation and also the complete virtual “dissection” and analysis of the vascular bed. Described as a graph, the vascular network provides a new tissue anatomy description, similar to city maps built on a road graph, showing structural clusters emerging as tissue “districts”. These clusters provide a new basis for lobule description in fat tissue. Even more can be obtained from virtual perfusion simulated inside the tissue by using in-silico modeling tools. This virtual perfusion reveals the functional properties of the vascular network, such as perfusion fluxes, and exchanges between clusters and infusion time (time for the blood to reach a site from the principal entry).

## Introduction

A better understanding of organ function and dysfunction requires a better knowledge of multiple, complex, dynamic and spatially organized cell interactions inside the whole organ. These interactions largely depend on the local oxygen tension and the supply of substrates brought by microvasculature. All these elements define a set of interacting heterogeneous micro-environments that are usually poorly characterized. Numerous works have used imaging to identify and define such micro-environment individually, but the global map of these subsets at the scale of the whole organ is most often lacking. However, a fine understanding of integrative liver functional anatomy at cell resolution has shown the interest of such an approach [1, 2]. This lack of whole-organ mapping is due to several technological bottlenecks ranging from imaging to the treatment of large datasets. Indeed, achieving the description of the micro-environment at the scale of the whole organ required the ability to image the whole tissue in its depth at cell resolution, then digitizing and vectorizing the large dataset corresponding to the image and finally to find a way to manage the dataset in order to draw the map and model of its functioning.

The use of various microscopy techniques has led to considerable recent [3–11] and opens the door to reconsider the functional anatomy of large tissue volume. Indeed, vascular structures provide structural information that is spatially extended (thus non-local) and graph-based (hereafter graph refers to the graph-theory object), directly relevant to perfusion and metabolic exchanges, for which an *in silico* tissue-scale analysis can bring significant understanding to tissue functions. Various studies have used large tissue imaging particularly in the context of brain perfusion analysis [5–7, 12–14]. Furthermore, large-scale organization analysis has already been pursued in the context of brain connectivity [15] to map functional couplings between various areas. These studies infer structure/function properties of a given tissue from the combination of cellular and micro-vascular imaging as well as perfusion modeling.

Recent developments in tissue preparation for the light-sheet fluorescence microscope (LSFM) (i.e. clearing, staining, labeling [16–19]) are now offering new prospects for deciphering supra-cellular organizations in tissues and revisiting the vasculature and functional anatomy of large tissue volume. The technique provides the ability to segment, detect, and quantify biologically relevant structures and patterns, especially vessels [11, 20–22]. These recent developments also raise new challenges for digitizing and treating massive 3D data.

A first step was carried out by Kelch et al. [4], who analyzed the lymph nodes with a dedicated and precise imaging of the microvascular structure as well as the topological organization, albeit on a rather limited sample size. To further tackle the challenge of imaging whole tissue, the inguinal fat pad is a convenient (and relevant) environment and of major interest in biomedical research.

Besides being easy to use and accessible, the fat pad can be cleared to image the whole tissue. In addition, its key role in the pathophysiology of metabolic disorders associated with metabolic regulation and energy storage crucially depends on perfusion [23, 24]. In this paper, we present a multi-disciplinary, image-based, blood-flow perfusion system to model a whole-organ (fat pad) vascular network for analyzing both its structural and functional properties. We provide significant steps forward in three directions: 1) we describe how an entire microvascular network of a large tissue can be imaged, segmented and reconstructed for a volume a hundred time larger than that examined in previous studies [5, 13], 2) we examine the functional role of microvascular perfusion by blood-flow modeling in the resulting network with 1.7 millions of vessels, and 3) we analyze the structural and functional organization of the tissue by using network clustering techniques adapted to this biomedical context. From these advances, a new picture of functional anatomy emerges, the significance of which is provided in Discussion.

## Material and methods

### Tissue preparation

**Fig 1:**
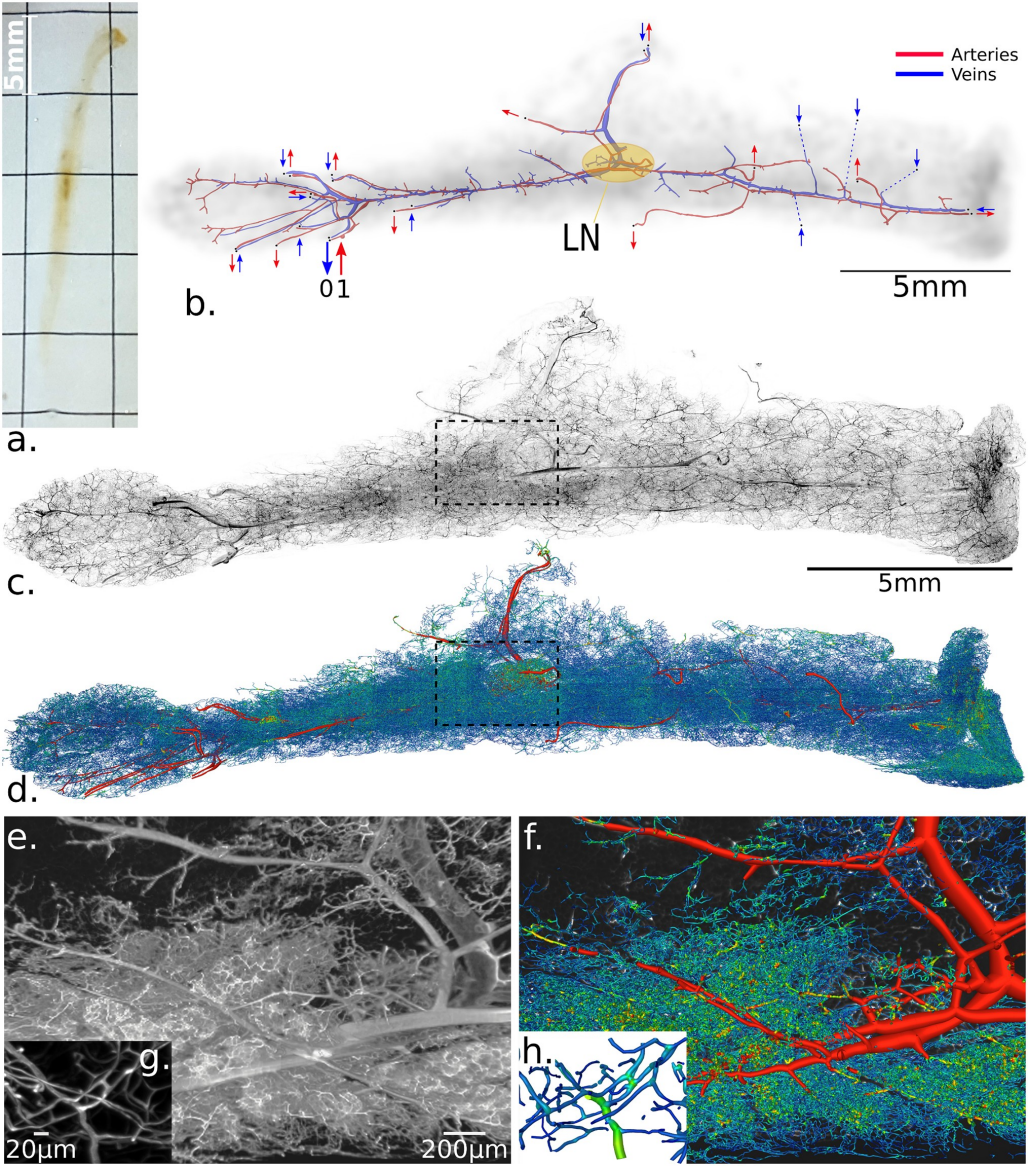
Original image and reconstructed network visualization. (a) The cleared fat pad tissue sample rotated vertically. (**b**) Schematic representation of the macrocirculation: arteries (in red) and veins (in blue). Large red arrows indicate the flow directions associated with the main feeding artery inlet into which (dimensionless) pressure *p* = 1 pressure is applied. The nearby large blue arrow also indicates the main draining vein outlet, with (dimensionless) pressure is set to *p* = 0. All others secondary vein inlets and artery outlets of the network are indicated with smaller arrows. The location of the central lymph node (LN) is illustrated by the yellow ellipse. Details of boundary conditions can be found in S5 Text. (**c**) Maximum intensity projection of the full fat pad tissue imaged with an LSFM, the volume of which was ∼ 25 × 5 × 1 mm^3^. (**d**) The vascular network extracted from the image (c) is based on image deconvolution, segmentation and vectorization. The vascular graph has ∼ 1.7.10^6^ edges and ∼ 1.2.10^6^ nodes (cf. Fig 2a and 2b for definitions). (**e**) Volume rendering of a small part highlighted in (c) for a small range of slices (for a total of ∼ 250*μ*m width) with, (**f**) the corresponding reconstructed vascular graph. (**g**) and (**h**) are higher zoom levels of (e) and (f), respectively, highlighting the high resolution (0.96*μ*m voxels width) needed for micro-capillaries segmentation.

The mice under study (6- to 8-week-old male C57BL/6J mice [Harlan Laboratories] on a 12-h light/dark cycle with free access to food and water) were injected in vivo with retro-orbital rhodamine-red–labeled Griffonia (Bandeiraea) and Simplicifolia Lectin I (Eurobio Abcys) to achieve proper vessel labeling. At 30 min after in-vivo lectin injection, animals were anesthetized by intraperitoneal injection of a ketamine/xylazine mixture and perfused intracardially with 4% para-formaldehyde solution, then tissue was removed, oriented and post-fixed overnight at 4°C. Tissue was embedded in 1% agarose before being dehydrated by ethanol, then cleared by incubation in benzyl alcohol-benzyl benzoate solution (BABB, 1:2 vol:vol ratio) (Sigma Aldrich). A clear fat pad is illustrated in Fig 1a. All experiments complied with European Community Guidelines (2010/63/UE) and were approved by the French ethics committee.

### Light-sheet Fluorescence Imaging Microscopy (LSFM)

Cleared samples were imaged on a custom LSFM based on cylindrical lens illumination with 488- and 561- nm lasers (Oxxius, France) and horizontal macroscope detection. The lens for the formation of the light sheet was an f = 50mm cylindrical lens coupled with a slit aperture of maximum 7mm opening to control the thickness and flatness of the illumination beam. The resulting thickness of the light-sheet at the waist varies from 5 to 50μm depending on the slit aperture. Thickness (*T*) was set to 35 μm to fit the largest field of view (FOV) at the lowest magnification (i.e., 1.2X). The macroscope was a Nikon AZ100M macroscope with an air lens of 2x magnification, providing an 8-fold zoom factor. The final magnification of 6.72x used here yields an estimated 1.83 μm lateral resolution against an axial lightsheet extent of about 20μm. Imaging in clearing media was through a 2 mm glass wall of a polished cuvette. Rotation of the sample was performed from below the sample, acting on the agarose block embedding the sample, with a driving rotation stage (M116-DGH, Physik Instrumente, Karlsruhe, Germany). Detection was performed with 50nm band-pass filters mounted in a filter wheel (LB10, Sutter Instruments, Novato, CA, USA) and a SCMOS camera (ORCA Flash4.0, Hamamatsu, Japan). Fig 1e illustrates the typical image of avascular network with voxel size of 0.967 × 0.967 × 2*μ*m. The full tissue acquisition consisted of 28 tile images of 1500 × 2048 XY voxel resolution with variable depth (Z dimensions) from 400 to 1500 voxels, for a total of ∼120GB raw 16-bit data. These tiles are stitched as described in [25].

### Network extraction

First, tile images were interpolated in the Z direction with cubic splines so that voxels matched an isotropic dimension of 0.967*μ*m width and were then subdivided into 256^3^ voxel sized blocs that could be processed simultaneously. Image deconvolution was then applied. The Richardson-Lucy (RL) algorithm was used to remove artifacts from light microscopy (e.g., [26]) and has been found appropriate and accurate for LSFM deconvolution [27, 28] although computationally challenging. Without the use of beads, the efficient estimate of the point spread function (PSF) becomes challenging, especially when its shape varies for spatially inhomogeneous light-sheet depth. Because we chose a large light-sheet depth and FOV, we considered a spatially homogeneous PSF designed with a Gaussian shape for which its standard deviation *σ*(*z*) = 11*μ*m is related to the light-sheet depth (cf. section Light-sheet Fluorescence Imaging Microscopy). More information about the choice of the PSF can be found in [29]. We then used the RL iteration updates with a total variation regularization. Deconvoluted images were then simply thresholded, and the resulting binary images were used for network extraction. The spatial graph representing the vascular network was built according to the vectorization of the binary image as described in [30]. Vessel center lines were extracted with an appropriate homotopy preserving skeletonization method [30]. The graph *G*_*v*_ = (*V*_*v*_, *E*_*v*_) was then created, where *V*_*v*_, the set of nodes, was extracted from the branching-voxel and end-voxel, and *E*_*v*_, the set of edges, was between connected node-voxels of the vessel skeleton. An edge consists of a chain of center-line voxels comprised between two nodes. Vessel radii were estimated following [31]: for each segment-element and node (illustrated in Fig 1b), a sphere was expanded until 10% of its volume was left outside the vessel shape in the binary image. Fig 1e illustrates the network extracted from Fig 1d with tubular-like visualization in Avizo (FEI) software. Once each of the 256^3^ voxels blocs was vectorized, networks were stitched according to global coordinates found in the previous step and merged with a simple coupling procedure to obtain the full network. Loose ends were connected by four successive Tensor Voting (TV) iterations with different parameters, trying to connect small gaps at first, with 5μm as a reference distance and 30° as reference angle (cf. [30] for more details) and up to 30μm as reference distance and 45° at fourth iteration. The connections added represented ~5% of the total vascular network edges. The quality of the skeletonization and reconnection procedures was quantitatively assessed in [32].

The above-described image processing is effective only on vessels and not macro-arteries and veins because they present different morphologies and responses to the lectin labeling. Capillary vessels (< 50μm) appear as filled near tubular structures, whereas arteries and veins appear as non tubular empty structures. A few studies tackled the particular case of automatic segmentation of such structures (e.g., [33, 34]), but we chose to manually segment these macrovessels to ensure a reliable vascular tree and geometries, which are essential for realistic flow simulations. Finally, the macrovascular network (illustrated in Fig 1) was merged with the microvascular network by adding a junction vessel by using the tensor voting method [30] as shown in Fig 1e, 1g and 1i. The resulting network was also carefully inspected along all large vessels to check and verify the quality of the reconstructed network versus the initial grey-scale image. This verified, all topological constraints needed for the modeling (Euler relation between vertex-edge number, no self-loop of segment elements, etc.).

Let us also define various notations from the graph *G*_*v*_. We denote its adjacency matrix ***A***, its degree matrix $D_{ii}=\sum_{j=1}^{N}A_{ij}$ and the Laplacian matrix ***L*** = ***D*** − ***A***.

### Edge and network resistance

The aspect ratio (radius divided by length) of vessels is small, on average (≃ 1/8, on average), which justifies use of a lubrication-based discrete network approach (cf. section Edge and network resistance). The previously described vectorized network allows us to compute the hydrodynamic resistance of each vessel, defined as the ratio of the flow rate to the pressure drop applied in the vessel, formally the analog of the resistance in electric circuits. The Hagen-Poiseuille law provides the relation between the resistance *R*, the flow rate *Q* and the pressure drop Δ*p* where the resistance is a function of the viscosity *μ*, the diameter *D*.

$$\begin{array}{c} -\frac{\partial p}{\partial s}=R\left(s\right)Q=\frac{\pi }{128}\frac{\mu (s)}{D^{4}\left(s\right)}Q \end{array}$$ (1)

To account for the effect on red blood cells to blood rheology, empirical models have been proposed to described blood viscosity, first *in vitro* [35, 36], then *in vivo* [37] and more recently *in vivo*, taking into account the effect of the endothelial surface layer [38]. In this work, we used the latter scenario because in a comparative study, it was found to have the most relative influence on resistance, pressure distribution and flow rate [13]. We also used an integrated formula of resistance because it considers the local shape variations of the vessel. Resistance in each vessel is thus defined as

$$\begin{array}{c} R=\frac{\pi }{128}(\int \frac{\mu (s)}{D^{4}\left(s\right)}ds{)}^{-1} \end{array}$$ (2)

The expression for viscosity *μ* can be found in [38, 46]. Given the resistance on each edge of the vascular network, one can define the network resistance between every pair of vertices. Based on [39], the resistance *R*_*ij*_ between two vertices of index *i* and *j* is

$$\begin{array}{c} R_{ij}=\sum_{k=2}^{N}\frac{1}{\lambda_{k}}{\left|\psi_{k,i}-\psi_{k,j}\right|}^{2} \end{array}$$ (3)

where λ_*k*_ and *ψ*_*k*_ are respectively the eigenvalues and eigenvectors, respectively, of the Laplacian ***L*** of the graph *G*_*v*_ for which the adjacency matrix ***A*** is weighted by vessel conductance.

### Graph clustering

Graph clustering, also called community partitioning, aims to regionalize networks [40]. We used a previously described method optimized for a large network [41]. The method maximizes the community features reflecting the density of edges between vertices inside communities as compared to edges between vertices in different communities. Considering vascular networks, clustering can be applied to the unweighted graph *G*_*v*_, denoted *G*_*v*_ = (*V*_*v*_, *E*_*v*_, *w*_0_) as defined in section Network extraction, as well as an edge-weighted version of *G*_*v*_, denoted *G*_*v*_ = (*V*_*v*_, *E*_*v*_, *w*_*i*_), where *w*_*i*_ is the weight function. We investigated four different edge weights, that could be of interest for vascular graph analysis: *w*_1_ the Euclidean distance between the two vertices connected by an edge, *w*_2_ the geodesic distance between the two vertices connected by an edge, *w*_3_ the hydrodynamic resistance of an edge and *w*_4_ the hydrodynamic conductance. Once the vascular graph *G*_*v*_ was clustered, we defined a new abstraction level of the vascular network denoted $G_{c}^{w_{i}}=\left(V_{c},E_{c}\right)$ as the adjacency graph of the communities found from *G*_*v*_ with the weight function *w*_*i*_. *V*_*c*_ thus represents the communities and *E*_*c*_ the connections between communities.

### Graph features

Graph theory is widely used to characterize structured networks in technological and transportation infrastructures, social relations, or biological systems (e.g., [15]). Here we focused on characterizing the effect of communities inside graphs, $G_{c}^{w_{i}}$, defined in section Graph clustering. We used two features: 1) the eigenvector centrality, which is a modified version of the betweenness centrality [42] and gives high scores for vertices playing the most central role (i.e., those with the smallest farness from others), and 2) the Authority/Hub features designed in [43] to discover authoritative and hub web pages on the World Wide Web concerning a topic search.

To analyze the arterial/venous vascular organization on both the network and the community scale, we computed arterial/venous domains based on generation numbers starting from the arterial and venous macro-vascular networks (Fig 4g). Both of these macro-networks were extracted from the full reconstructed network by traversing the vascular graph from every arterial boundary condition, and also from every venous boundary condition, under the condition that the mean vessel diameter is ≥ 15*μ*m. Then, starting from the vertices of the arterial macrovascular graph, we increased by one the generation number at severy vessel connected to those nodes. The corresponding vertices of the vessels found were then looped on. Numbers were further increased by one during each loop. This procedure was continued until no further vessel was found. The very same algorithm was used starting from the venous macrovascular graph. Because of the proximity of the arterial and venous macrovascular networks, the generation numbers from both networks were very similar (see S4 Text). Finally, we used the following criterion to decide whether a vessel was part of the arterial or venous domain: if its generation number starting from the arterial macrovascular network was greater than its generation number starting from venous macrovascular network, it was considered part of the arterial domain. Otherwise, the vessel was considered part of the venous domain. At the community scale, we then computed the fraction of vessels that were part of the arterial domain. The result is displayed in Fig 4g.

### Flow modeling

The adopted discrete perfusion network is based on previously proposed microcirculation models [44, 45]. These frameworks were previously used to model blood perfusion from image-based network extraction [5, 12, 13]. Here we describe the main steps and modeling issues.

The flux conservation applied at any vertex *i* with the set of its neighbor vertices is denoted as *J*

$$\begin{array}{c} \sum_{j\in J}Q_{ij}=0 \end{array}$$ (4)

where *Q*_*ij*_ is the flow on an edge connecting vertices of *i* and *j*. From considering an integrated local lubrication approximation ([13] for more details as well as reference therein), one can provide a relation between the pressures at every vertex, which are is not associated with boundary conditions

$$\begin{array}{c} \sum_{j\in J}C_{ij}\left(p_{i}-p_{j}\right)=0 \end{array}$$ (5)

where *C*_*ij*_ is the conductance of edge connecting vertices *i* and *j* and *p*_*i*_ (resp. *p*_*j*_) the pressure on vertex *i* (resp. *j*). For every other vertex, a pressure Dirichlet boundary condition is imposed whose values will be discussed later.

Applying these relation to every vertex of the graph gives the following system

$$Lp=b_{D}$$ (6)

where ***L*** is the Laplacian matrix of the vascular graph *G*_*v*_ with hydrodynamic conductance as a weight, ***p*** is the vector of pressures to solve and $b_{D}$ is the vector of applied boundary condition, which is zero whenever the index is an internal vertex and $p_{D}\cdot C_{ij}$ whenever the index is a boundary condition.

The common framework for blood-flow network simulations considers also the variation of hematocrit. The hydraulic conductance (or resistance) being a function of hematocrit, this results in a coupled system of equations. For converging bifurcation, the mass conservation law gives a set of equations and for diverging bifurcations, empirical models give the fraction of hematocrit that will be convected in both daughter vessels. Reference [46] shows that hematocrit variation has a small effect on blood perfusion. Thus, we considered a constant hematocrit, with a value of 0.45 throughout the network.

A pressure Dirichlet boundary conditions is associated with any identified vertex corresponding to a cut-open macrovessel edge. A careful analysis of the network architecture allows for exhibiting a total of 26 macro-vascular vessels entering and leaving the tissue. Thus, 26 boundary conditions must be set for the flow modeling (see section Flow modeling for details). We found a total of 26 macro-vessel edges, as illustrated in Fig 1a. Expert knowledge of the macrocirculation of adipose tissue reveals that only one main inlet artery perfuses the tissue, the location of which is known in this tissue (large red arrow in Fig 1a). Similarly, because the feeding and draining vessels are associated in pairs, the corresponding vein is thus located (large blue arrow in Fig 1a). The dimensionless pressures associated with these two main inlets/outlets are defined as 1 and 0. Then every other boundary condition is considered as a secondary outlet artery or a secondary inlet vein, as shown in Fig 1a. This feature is generic to whole organ imaging because there are always various macrovessels in a given tissue, that are unavoidably cut. To reduce the problem complexity, we imposed the same inlet vein boundary condition (denoted *α*) and outlet artery boundary condition (denoted *β*) to every secondary pressure boundary condition. To reduce the impact of perfusing inlet veins, we computed the relative perfusion volume (relative to the perfusion domain) of the main inlet artery over the sum of all perfusion volumes of every inlet vein. We chose *α* and *β* values to maximize this ratio, which resulted in *α* = 0.35 and *β* = 0.3125. S5 Text summarizes the detail of perfusion volume ratios for a large range of *α*, *β* values. Perfusion domains are sub-graphs of the micro-vascular network, providing, from each entry, its (predominent)) basin of perfusion inside the entire network. In brief, it is evaluated by using a spanning-three method, starting from each entry, but restraining the sub-three extraction to predominant flux branches at each bifurcation. For more details concerning the definition and computation of perfusion domains, refer to [14].

We also investigated the effect of diameter variations that might occur due to segmentation errors on blood flow exchange between communities. Overall, a few pairs of communities ceased to exchange blood and the rest did so with a relative quadratic error up to 40%. More details can be found in S8 Text.

## Results

### Tissue organization both at the macro and micro-circulatory scales

**Fig 2:**
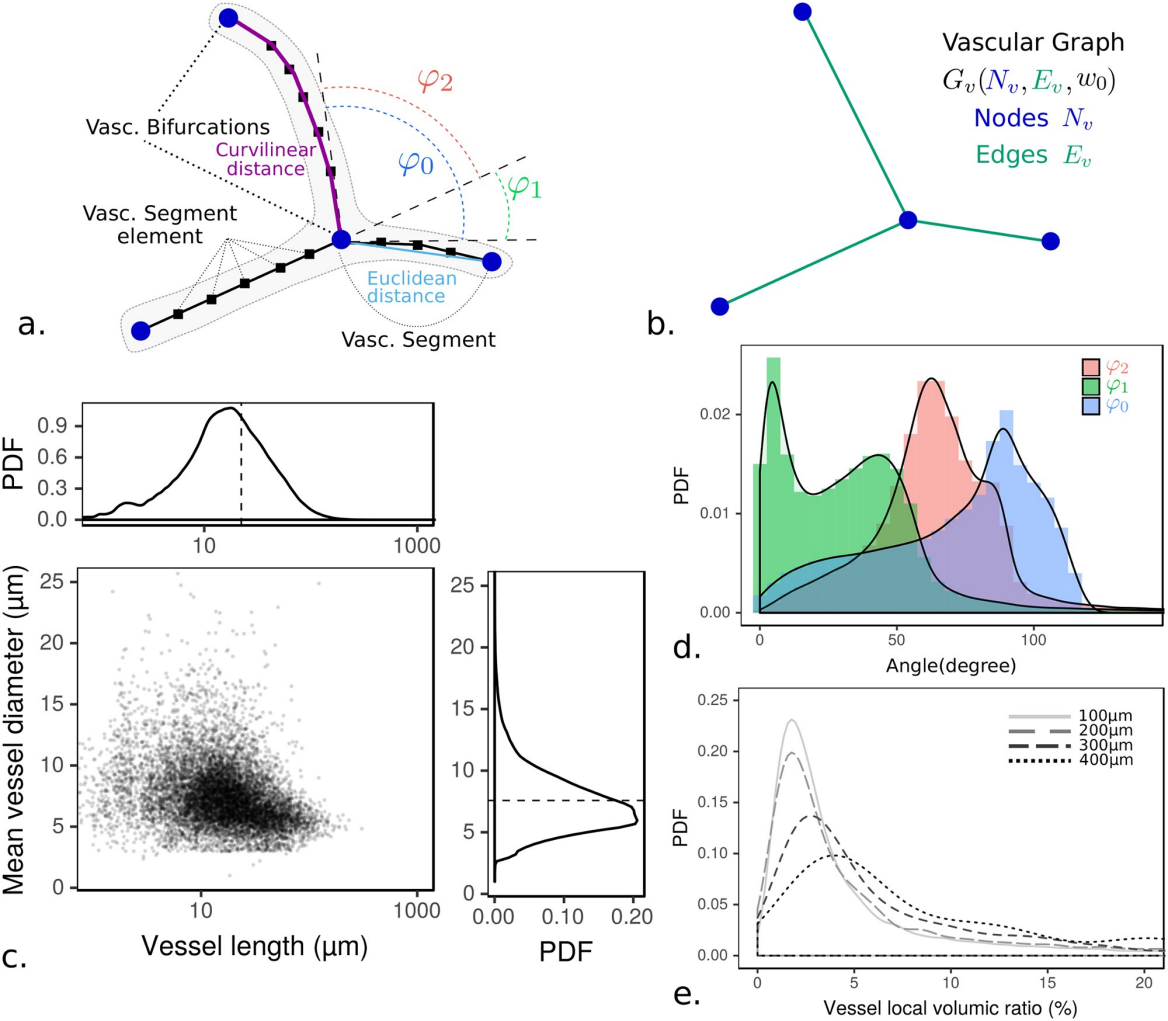
Vascular network statistics. (**a**) Schematic representation of the vectorized vascular network and (**b**) the corresponding vascular graph with common key concepts in both cases. Note that vascular segments are turned into graph edges when the vascular network is mapped onto its graph representation. (**c**) Data for vessel length and diameter. The average aspect ratio is found equal to *D*/2*L* ∼ 1/8. (**d**) Histogram distribution of bifurcation angles (cf. Fig 1b for angle conventions). (**e**) Local vascular density (volumetric ratio) distribution measured in the whole network according to four elementary box size widths from 100*μ*m to 400*μ*m.

The macrovascular network (see section Network extraction for details about segmentation) extracted from the inguinal fat pad (Fig 1a and 1b) follows the known main anatomical architecture of the tissue: a principal longitudinal (apex-groin) axis, bifurcating nearby the lymph node into a (upside-down) ‘T’ shape that leads to the major arterial and venous pair [47]. The main macro-vascular artery inlet, associated with a main vein outlet, is located on the left of the tissue and is responsible for the main perfusion. Image acquisition of the whole tissue (Fig 1c) and segmentation of its entire vascular network (Fig 1d) allow for quantifying microvascular networks at close to micron-scale resolution, that is, with several voxels per diameter (Fig 1e, 1f, 1g and 1h), with their associated local parameters (diameter, segment length, orientation, density, etc.). Indeed, these structural local parameters allow for a vectorized description of the vessel (Fig 2a) and the corresponding vascular graph (Fig 2b). In contrast to many other contexts in which graphs are found in nature (e.g. metabolism pathways, gene interactions, ecology, etc.) or in practical applications (web-structure, social web, etc.), the local structure of the graph *G*_*v*_ of microvascular networks is very simple: almost all bifurcations result from the connection of two distinct vessels into a single one or vice-versa (Fig 1g and 1h). The vascular graph can be manipulated by associating different graph weights *w*_*i*_ (e.g. *w*_0_ as illustrated in Fig 1b), that is the scalar quantity associated with each edge connecting two nodes (i.e. bifurcations) which is provided in order to consider different levels of relationships, weak or strong, between elementary nodes.

Considering this framework, one can quantify key aspects of the micro-vascular network. The aspect ratio (radius, divided by length) of vessels is small in average (≃ 1/8, Fig 2c), so as to justify the use of lubrication-based discrete network approaches (see Material and methods section Flow modeling). The segment’s orientation distribution at bifurcations given in Fig 2d displays two predominant configurations: a T-shape associated with quasi-aligned daughter and parent branches (first mode of *φ*_1_ distribution and second shouldering of *φ*_2_ one), and a Y-shape family one (second mode of *φ*_1_ distribution, first mode of *φ*_2_).

Now, considering the entire tissue, we first evaluated the vascular density inside boxes of varying sizes (from 100*μ*m to 400*μ*m, Fig 2e, as in [29]). This evaluation produced variable density distributions without the emergence of a homogeneous length-scale and thus revealed strong heterogeneity. Similarly, the hydraulic resistance (see Material and methods subsection Edge and network resistance and S1 Text) and the pressure distribution inside the vascular network (S1 Text) display various heterogeneous scales. Hence, various vascular characteristic scales emerge from our analysis, considering both geometrical properties (i.e. vascular density) and hydrodynamic ones (i.e. hydraulic resistance and pressure distribution), the presence of which highly suggests a meso-scale organization that we next investigated.

### Structural units emerging from clustering analysis

Meso-scale vascular organization results from clusters of vessels related by a close proximity (either from structural or functional relations). Graph clustering is an established technique mostly used in computer science for applications in web networks, social networks, etc., which allows for deciphering preferential information transfer between meso-scale entities. We adapted it here for our vascular network as in [5].

At the local scale, the minimum unit of the vascular graph is a simple local binary tree (Fig 1b), but at a broader scale, this graph is much more complex, involving preferentially connected units called communities (Fig 1f and 1h). Clustering methods are based upon finding a graph partitioning (i.e. a separation of the graph into non overlapping units) that maximizes the density of (weighted) edges between vertices inside communities as compared to edges between vertices in different communities (cf. subsection Graph clustering). Hereafter we refer to the result of the clustering partitioning of the vascular graph as communities.

**Fig 3:**
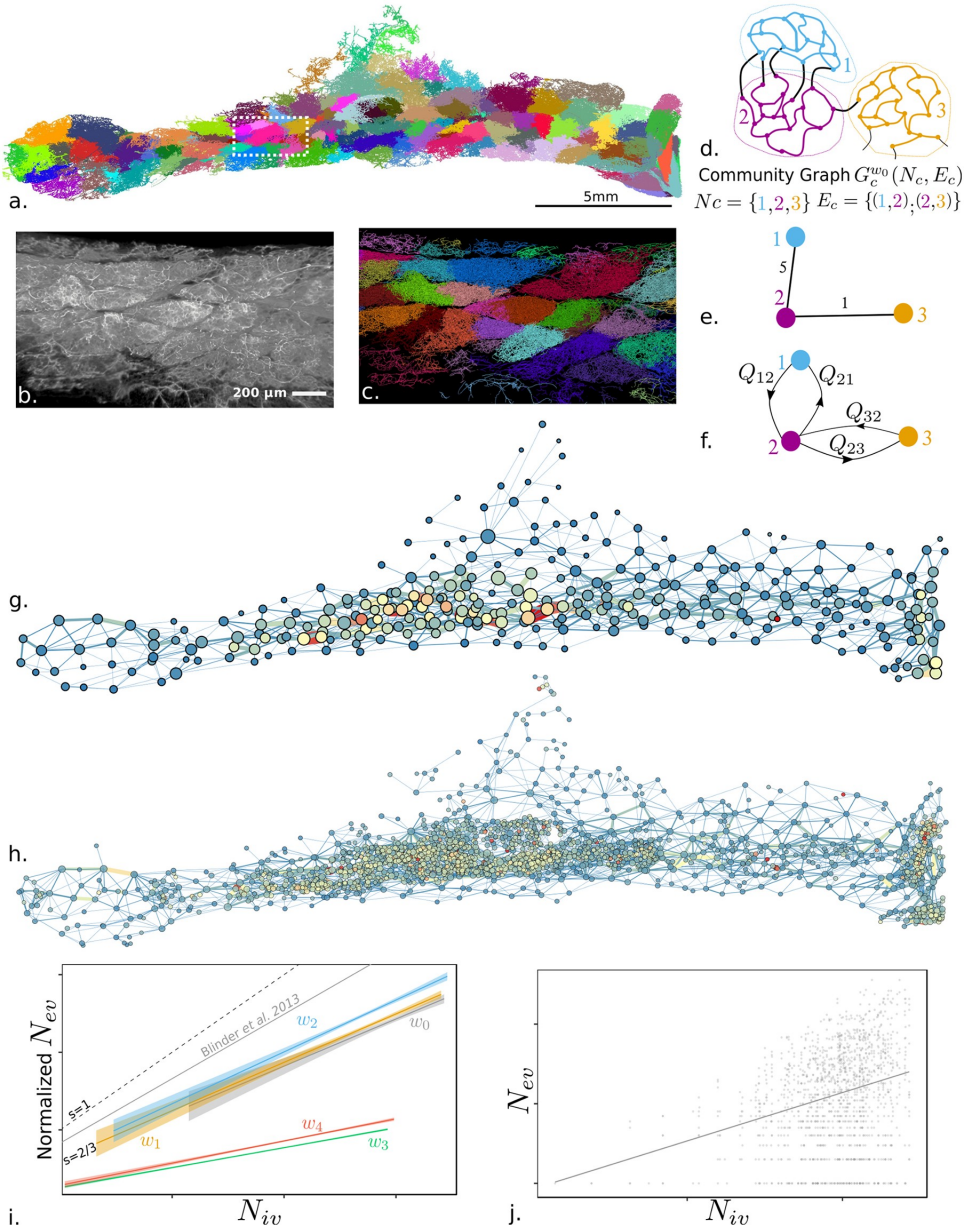
Vascular graph clustering. (**a**) Community clustering applied to the vascular network by using an unweighted vascular graph representation, *w*_0_. Color codes for community membership. (**b**) and (**c**) Image volume rendering and the vascular graph segmentation, respectively, of a sub-volume (∼100μm width) extracted in a dense and modular area of the tissue near the central lymph node (white dotted rectangle depicted in (a)). (**d**) Schematic illustration of vascular communities; (**e**) structural community graph, with the number of vessels connecting two communities as edges weights (5 and 1 here); and (**f**) bi-functional community graph with in/out fluxes *Q*_*ij*_ as edges weights. (**g**) Graph clustering of the communities for geometrical based weight (*w*_2_, curvilinear distance) and (**h**) perfusion based weight (hydraulic resistance *w*_3_); the disks are located in community centers projected in the (*x*, *y*) plane with sizes coding for volumes and color for vascular density (volumic ratio from 0% to 20%) illustrated with a cold-to-warm color scale. (**i**) Linear regressions of the scatter plot of number of vessels between each pair of communities *N*_*ev*_ versus number of vessels in the community *N*_*iv*_ (for each community in (d), number of colored edges vs. number of black edges). Full lines are the linear regressions for each clustering weight *w*_*i*_. *w*_0_ no weight (slope s = 0.61); *w*_1_ Euclidean distance (cf. Fig 2(a)) (*s* = 0.62); *w*_2_ curvilinear distance (cf. Fig 2(a)) (*s* = 0.66); *w*_3_ hydraulic resistance (Cf. S2 Text) (*s* = 0.25); *w*_4_: hydraulic conductance (Cf. S2 Text) (*s* = 0.28). The gray curve is the result of Blinder et al. [5] with *s* = 0.83. Dashed lines delimit the boundaries of weak communities (therefore having strong connections between units) with slopes *s* = 2/3 and *s* = 1. Shaded areas are the standard errors associated with each regression. The linear regressions are normalized with zero offset at the origin. (**j**) Non-normalized scatter plot for weight *w*_0_ with the associated linear regression in a solid line (other weights are reproduced in S2 Text).

We consider two classes of geometry and perfusion based family for weights *w*_*i*_ associated with vascular segment edges: class 1, structural associated with *w*_0_, *w*_1_, *w*_2_ for topological connection (e.g. Fig 2b), Euclidean distance (Fig 2a) or curvilinear (Fig 2a) respectively; and class 2, a perfusion-related relation associated with *w*_3_ and *w*_4_ for the vessel’s resistance and conductance respectively. For each weight a different set of communities is obtained, with one result for weight *w*_0_ exemplified in Fig 3a, 3b and 3c. We first focus on the results obtained with the simplest weight, *w*_0_, for which the relation between entities is binary (1 for a connection, 0 otherwise).

Fig 3a illustrates the spatial extent of the communities, all having vascular segments with homogeneous color. A zoom inside a sub-domain of the whole organ shows how structural entities already appear to have variable density, as visualized by maximum intensity projection (Fig 3b). The resulting graph communities spatially mapped into the vascular network with a different color for each community (Fig 3c) closely following the shape of these visible structures. This observation illustrates and validates the relevance and interest of community clustering for detecting and isolating vascular structural entities. The community of vessels (Fig 3d) can be schematically represented as a graph, with adjacent communities linked by an edge coding the number of vessels (structural community graph Fig 3e) or in/out fluxes (bi-functional community graph Fig 3f). Such a structural community graph with weight *w*_2_ (curvilinear distance) is illustrated in Fig 3i, in which the position of center of masses (by projection in the 2D (*x*, *y*) plane), distances between communities, volumes, as well as vascular density are also coded, revealing spatial heterogeneity of the vascular density distribution inside the tissue. The large vascular density variations already revealed from the wide distribution of the vascular densities histogram of Fig 2e can now be much more precisely attributed to the presence of structural entities, the spatial distribution of which is neither regular nor patterned. A clear structural trend emerges from Fig 3g: the highest vascular densities (red and oranges circles) are found in a core region, nearby the trajectory of the main feeding artery (shown on Fig 1b). Fig 3h shows a similar trend for a perfusion based weight partitioning (*w*_3_ hydraulic resistance) with about 10 times more communities and a much denser central region in terms of community numbers. This larger number of communities found with functional weight is robust (less than 1% variations) when taking into account the local blood flow orientation found in the blood flow modeling, and performing the clustering onto the oriented graph with weight *w*_3_. Furthermore, the number of connected vessels associated with the edge sizes of Fig 3g and 3h reveals more heterogeneous couplings between structural communities (Fig 3g) as compared with the smaller and more homogeneous couplings for functional communities (Fig 3h). This difference between the two classes of weights suggests differences in interconnections of communities, which we next examined.

To evaluate the connectivity of communities, in Fig 3i and 3j, for each community pair, we computed the number of external connecting vessels *N*_*ev*_ (e.g., for the communities of Fig 3d, number of black edges) and plotted it against the number of internal vessels *N*_*iv*_ (e.g., for community #1 of Fig 3d, number of blue edges). The power law scaling of *N*_*ev*_ against *N*_*iv*_, such as the representation in Fig 3d, allows for delineating a zone of weak communities (i.e., strongly connected ones) when the slope of the scaling is between 2/3 to 1 (larger than a surface to volume ratio) and strong ones when the slope is smaller than 2/3 (i.e., weakly connected nodes). Additionally, strong communities are found when the slope of the scaling is less than 2/3 (both slopes shown in Fig 3i). Considering the first class of geometry-based weights (*w*_0_, *w*_1_, *w*_2_), we observe in Fig 3i that the number of vessels connected with external ones indeed scaled similar to the number of internal links *N*_*iv*_ of the community, in this case, $N_{ev}∼N_{iv}^{0.63\pm 0.03}$. With the lower bound scaling for weakly connected units being a 2/3 power law, our results indicate that structural clustering results in already quite strongly connected units. Considering the second class of perfusion-based weights (*w*_3_ and *w*_4_), an even more significantly different scaling is found (cf. Fig 3d), $N_{ev}∼N_{iv}^{0.27\pm 0.01}$, revealing very strongly connected perfusion communities. This very distinct behavior shows that clustering using structural weights (class 1: *w*_0_, *w*_1_, *w*_2_) or functional weights (class 2: *w*_3_ and *w*_4_) can reveal vascular units with distinct average densities and sizes (S3 Text). We then investigated how these structural communities are functionally coupled.

### Vessel community perfusion coupling map

Here we consider functional perfusion distribution between communities. We focused on the clustering results obtained from one structural weight (i.e., *w*_2_ associated with the curvilinear distance defined in Fig 2a) because structural weights provide the most anatomic-relevant information (compared to [47]). We analyzed the total perfusion exchange fluxes between a given pair of communities (depicted in Fig 3h) denoting $Q_{c_{i},c_{j}}$ as the total flux from community *c*_*i*_ to *c*_*j*_, that is, $Q_{c_{i},c_{j}}=\sum_{k\in K_{c_{i},c_{j}}}Q_{k}$, where $K_{c_{i},c_{j}}$ is the set of vessels connecting communities *c*_*i*_ and *c*_*j*_, flux *Q*_*k*_ being directed from *c*_*i*_ to *c*_*j*_ ($Q_{c_{j},c_{i}}$ is defined vice-versa). The robustness of the perfusion modeling and total flux exchanges between communities to the quality of the segmentation and possible errors in vessels vectorization is provided in S7 Text.

**Fig 4:**
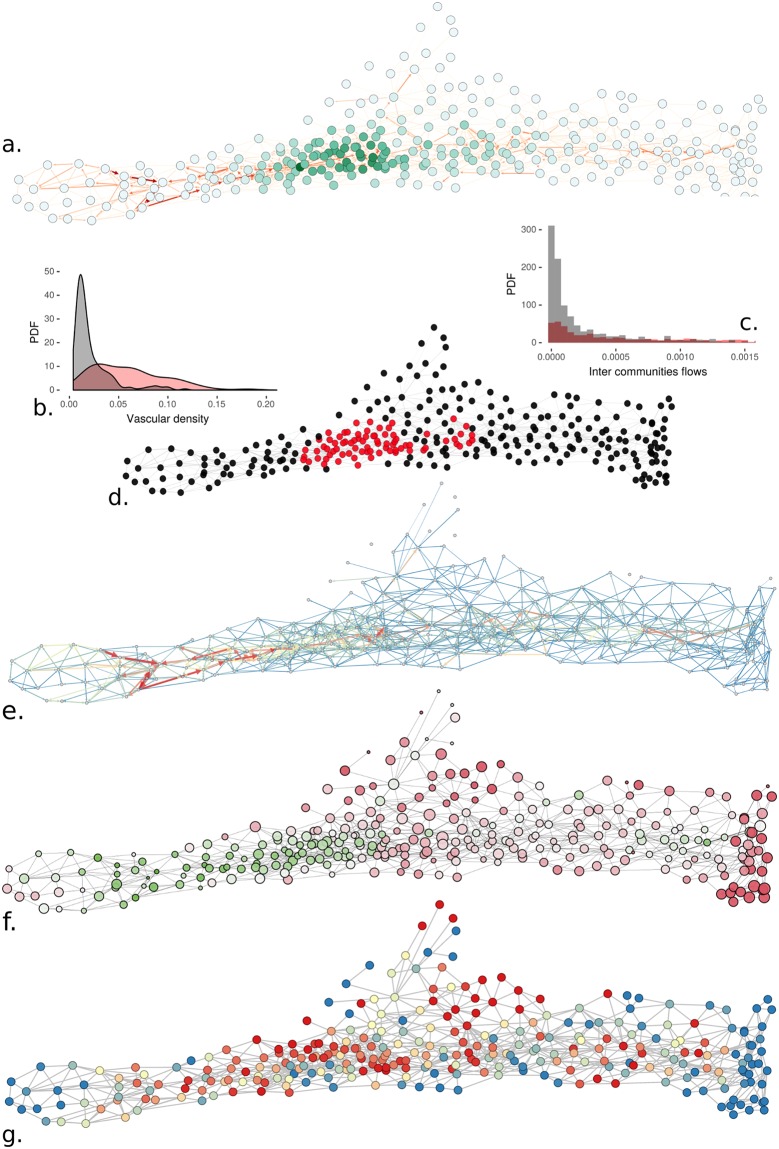
Functional community network analysis. (**a**) Graph authority feature on the bi-functional (cf. Fig 3g) communities graph. The color code, from white to dark green indicate small to large graph authority value. (**b**) Identification of central area by selecting nodes with the authority graph authority feature (>0.04) core in red and (<0.04) peripheral in black. (**c**) Histogram distribution of vascular densities in the region depicted in (**b**) using the same color-code (mean vascular density is 0.06 for red, 0.02 for black), Kruskal-Wallis rank sum test: p-value < 0.0001. (**d**) Intercommunity flows of region depicted in (**b**) using the same color-code. Mean flow inside (red) is 0.0079, outside (black) is 0.0212, Kruskal-Wallis rank sum test: p-value > 0.05. (**e**) Bi-functional community graph (as defined in Fig 3g) with edges colored and sized by the perfusion fluxes (two arrows per edges for in/out fluxes not always visible due to fluxes asymmetry). (**f**) Dynamics of perfusion at the community scale. The nodes are sized according to the relative filling time of each community and are colored according to the fluid global time arrival (with a green-to-red scale) with green nodes fed first and red ones last. (**g**) Arterial domains computed from generation numbers from blue to red from 0 to 100% (100% means all vessels are closer to arterial macro-vascular network than to veneous one).

Computation of graph authority (cf. subsection Graph features for definitions) associated with functional graph features (Fig 4a) reinforces the identification of a core region previously found for structural properties (Fig 3i and S4 Text). Thus, blood perfusion confirms the spatial organization of the graph communities. Furthermore, Fig 4a, 4b, 4c and 4d shows that the core region strongly co-localizes with the largest perfusion exchanges where macrocirculation vessels illustrated in Fig 1b are also present.

The perfusion dynamics (cf. S6 Text for more details) emphasize the significance of core regions of the core region prerogatives, because Fig 4e and 4f shows that these central communities are fed first, but also present heterogeneous filling times, that is, the time for a community for its entire vascular bed to be perfused from the arterial entry. Also, similar heterogeneities of filling times are observed outside the core region. This observed heterogeneity is related to the distinct and variable proximity of each community with the principal arterial trunk.

Finally, one can also analyze the communities by their ability to drain perfusion from the main arterial/venous trunk. To investigate this, we compute the arterial/venous domains based on generation numbers starting from the arterial and venous macro-vascular networks (Cf S4 Text). In Fig 4g we represent the fraction of vessels that are closer (in terms of generation numbers) to the arterial than venous trunk. The core region already depicts a clear proximity to the arterial trunk. This finding is consistent with the previous observations for this section, all flow-related.

## Discussion

Organ-scale supra-cellular exchanges represent crucial complex systems and have been poorly studied. In this study, we show a sensible step forward in this direction with extensive analysis of detailed perfusion at the tissue scale. Indeed, microvascular imaging, post-processing and modeling present many challenges, associated with combining high-quality imaging, huge data processing, and dedicated modeling issues. In this paper, we show that these challenges can be overcome to provide an emerging picture of tissue structural and functional anatomy at a whole-organ scale.

First, combining specific tissue-preparation techniques (clearing and sample positioning) with a dedicated large-scale LSFM set-up [29] allowed for high-contrast vasculature imaging with nearly terabyte-sized image mosaics that match the challenge of full organ segmentation and analysis.

Second, we used image processing tools and pipelines (stitching, registration, deconvolution, binarization, skeletonization) with this massive volume of data for segmenting and vectorizing vessels, which resulted in a huge memory saving for the manipulation and analysis of micro-vascular networks of 1.7 million vessels. The use of such pipelines provides sensible structural measurements for the vessel’s geometries and shape, such as vascular density, vessel length, orientation, micro/macro separation, spatial location, and arterial and venous territories, thereby providing an unprecedented precise picture of local heterogeneities and couplings.

Third, we present how vessel vectorization can be sparsely manipulated within a graph network by using clustering analysis techniques to reveal both structural and functional (i.e., blood flow related) units.

Biologically, the approach yields significant results to better understand the 3D topology of organs. Two distinct—structural and functional—families of graph weights (*w*) were chosen and yielded two classes of consistent results. Structural clustering, leading to what we call structural communities, resulted in about 200 clusters, the largest also being the more connected inside the center of the tissue, in the proximity of the principal feeding artery. We consider now functional clustering, associated withfunctional communities. Functional clustering provides about 10 times more clusters, which are smaller and denser in the central region, with very rich inter-connections, and larger in the periphery, with much fewer connections. The average volume of these clusters (found from the distribution mode of the cluster *w*_3_ volume provided in S3 Text equal to 6.10^−4^*mm*^3^) corresponds typically to 23 adipocytes (estimated from the mean cell diameter of about 37*μ* (cf. S8 Text). The numbers of adipocytes per cluster are of the order of magnitude of 13 ± 6, the mean adipocytes number found in double labeling images (cf. S8 Text). Hence, functional clustering provides insights into the existence of an intermediate level of functional unit inside large structural clusters. The unit might be related to some vascular regulatory potential, as suggested by functional flow modeling. Hence, functional clustering reveals the existence of an unexpected organization scale inside large clusters. Consistent with our previous reports [47] [48], this finding clearly emphasizes the heterogeneity of adipose tissue and its multiscale organization particularly for the oxidative metabolism point of view that strongly depends on vasculature and perfusion.

Regarding the results associated with structural clustering, we found that determining the proximity between vessels allowed for confirming the presence of large, well-connected structural units. These anatomical/structural clusters are heterogeneous, but are located in the vicinity of the principal arterial trunk and the lymph node. Similar structural entities have been described [47] by using different imaging modalities (i.e., confocal microscopy and epifluorescent immunostaining). These entities have been found to concentrate adipocytes having discriminant browning (ability to turn into brown fat) potential [47]. The biological analysis of these entities was refined in [48] with micro-dissection and immuno-labeling showing different expression levels of browning genes and mitochondrial apparatus in the core region as compared with the periphery, but also strong heterogeneity in adipocyte browning abilities within the core of the fat pad. As found in [47] and [48], here we reveal a central region located near the lymph node where structural entities have been identified, and a peripheral region with no particular structural units. Both central and peripheral regions were again found in the clustering analysis described in the previous section, and some explicit examples of clustering segmentation allowed for distinguishing central and peripheral clusters. Furthermore, various structural graph properties (such as centrality and authority) were strongly correlated with local anatomic features (vascular density, vessel connections) as well as functional features (total blood perfusion and exchange flux perfusion time). The primacy of these vascular-based network parameters near the lymph node region is highly associated with the high browning-gene expression found in [48], thus corroborating the well-described relationships between vascularization and the browning process. Hence, we found a vascular-based body of evidence for the existence of a central, well-perfused, strongly–connected and, highly-coupled region of adipose tissue as opposed to the more unstructured, poorly-connected, peripheral organization. Although a similar conclusion was raised previously [47], our study provides significantly more quantitative, objective and functional evidence using a supplementary approach. One significant outcome of this contribution is to show that the previously found enhanced local browning ability of adipocytes cells coincides with preferential vascular fluxes and vascular network clustering properties. This observation could suggest that the density of the vasculature predicts the browning area. Furthermore this distinct structural regionalization was not obtained from a direct visualization of a targeted cellular function but emerges from a non-biased structural analysis of the micro-vascular organization itself. Therefore, it reflects an emergent structural property of the network. Because both local cellular functions and vascular structural organization provide similar qualitative patterns of organization, their biological and metabolic relevance is strengthened. These new results also demonstrate that the vascular density of clusters located near the central feeding macrovessels is much larger than in the periphery, the vessel volume inside some clusters being as large as 20% of the total volume. As compared with other organs such as the brain, with gray-matter vascular density being 2% to 3% in primates or rodents (cf. [29, 31] and references therein), such large vascular density is significant. A similar vascular density is seen in only a few specific organs such as the kidney or spleen [49]. Hence, these findings emphasize the relevance of microvascularization in the metabolic and endocrine roles of adipose tissue.

In a broader context, the relevance of graph-based analysis associated with tissue-scale vascular networks offers great potential for deciphering the presence of a micro-environment in tissues as well as inter-compartment exchanges. This area was previously investigated in the brain in the search for microvascular units [5], as possible echoes of neuronal units, such as barrels in the somatosensory cortex. Nevertheless, this study did not find strongly connected vascular communities in the cortex (i.e., no “graph-based vascular barrels”). This negative result might result from the effect of finite sample edges associated with artificial blood-vessel ends (i.e., free edges) resulting from physical sectioning of the organ. Being able to analyze the reconstructed vascular network inside an entire tissue to circumvent this issue sheds new light on organ functionalities. This analysis of adipose tissue revealed strongly connected vascular units, also showing heterogeneous multi-scale functional couplings. Many endocrine organs feature strongly localized specific metabolic functions. The possible coupling between these functions via perfusion is poorly known. Our graph-based vascular approach offers great potential to better estimate functional couplings and heterogeneities in these organs.

For the community clustering results associated with perfusion-oriented weight, we found similarities and differences depending on structural weights. Our results in the fat pad significantly differ from those in the brain [5], where no strong communities have been found: we show the presence of vascular communities, the structure of which are strongly correlated with perfusion modeling. Of note, the results of [5] consider a weight based on hydraulic resistance with a different empirical viscosity model. In contrast to structural weights, use of functional weights reveals new small-scale functional entities associated with a much smaller volume. Furthermore, these small clusters are strongly connected inside each other. This observation might have future implications because it might be related to tissue remodeling scenarios. Indeed, small-scale perfusion units could be versatile and flexible to structural changes for angiogenesis. These small-scale units being much more numerous inside the large central clusters might result from a preferential remodeling ability consistent with their attested browning potential. Similar remarks could be raised in the peripheral region, where small-scale clusters are much less dense and numerous, with no browning potential observed [47]. Thus, the paradigm of isolated lobules [47, 50] as autonomous and disjoint metabolic functional entities in adipose tissue must be clearly revised. Our analysis suggests rather various scales of communities that are clearly spatially and functionally distinct but strongly coupled. The physiological relevance of such multiscale organization clearly requires defining the metabolic specifities of adipocytes not only at the single cell level but also with respect to their location.

Considering the perfusion map estimated from modeling and analyzed within the structural clustering family, we note that the total perfusion fluxes entering or leaving any cluster entity (the input flow exactly equals the output flow because of blood incompressibility) provide a first estimate of the metabolic dynamism of each entity. Our study shows for the first time a very refined distribution and regionalized perfusion fluxes into the tissues.

As expected, we found that the central units associated with structural communities are indeed those with the largest fluxes. Moreover, considering structural topological parameters (centrality) in order to distinguish the center from the periphery, we found significantly different perfusion rates in the central region than in the periphery (cf. S4 Text). This observation is not so surprising because the central clusters are indeed located near the main feeding artery so that some clusters are directly provided with blood. Nevertheless, this is not the case for every cluster, so more complex flow patterns are responsible for the strong perfusion found in various areas of the central region. Indeed, flow modeling also reveals important visible local heterogeneities that have not been reported elsewhere. We also analyzed the metabolic coupling between structural communities considering the perfusion flux between them but did not find a significant difference in perfusion exchanges between communities in the central region as in the peripherical region: this observation could be paraphrased with the urban metaphor, with no difference in exchanges between poor and rich neighborhoods. Our observations in the mouse fat pad might be due to the strong heterogeneity in perfusion exchanges in both regions. Finally, when considering the perfusion time, i.e. the time for the blood to either reach or fill a given cluster, clearly the central region is first fed by the principal arterial trunk. Such result was totally unexpected because it was reasonable to believe that perfusion first occurs in white fat then brings substrates relased by white adipocytes to brown/beige adipocytes for their thermogenesis. The opposite order of perfusion gives rise to the issue of its physiological relevance, which needs further investigations but is out of the scope of this manuscript. However, we can speculate about a putative role of brown/beige adipocytes upstream of the fat pad to control oxygen tension and redox substrate supply to the white part, as recently proposed in [51].

### Conclusion

In conclusion, our study demonstrates the interest of new imaging and segmentation tools for analyzing structural and functional organization in tissue. We believe the presented methods as well as their technical improvements will be useful in many future biological and biomedical tissue-oriented studies. This work provides the proof of concept that in-silico all-tissue perfusion modeling can reveal new structural and functional exchanges between micro-regions in tissues, found from community clusters in the vascular graph. It also illustrates how the combination of state-of-the-art LSFM imaging, huge image data post-processing and mathematical modeling can be combined to reveal the intimate anatomy of tissues.

## Supporting information

S1 Text Functional related heterogeneities on full vascular graph. (PDF)

S2 Text Detailed results for all clustering weights. (PDF)

S3 Text Community statistics. (PDF)

S4 Text Further structural and functional analysis. (PDF)

S5 Text Details of flow modeling. (PDF)

S6 Text Kinetics computation. (PDF)

S7 Text Effect of segmentation errors on perfusion exchanges between communities of vessels. (PDF)

S8 Text Small adipocytes clusters versus functional clustering. (PDF)
