# Supplementary material for: From whole-organ imaging to in-silico blood flow modeling: A new multi-scale network analysis for revisiting tissue functional anatomy
Source: PLoS Comput Biol. 2020 Feb 14;16(2):e1007322. doi: 10.1371/journal.pcbi.1007322 (PMC7062279; doi:10.1371/journal.pcbi.1007322)
Supplement: S1 Text — (PDF) [file pcbi.1007322.s001.pdf]

SI 1 Functional related heterogeneities on full vascular graph

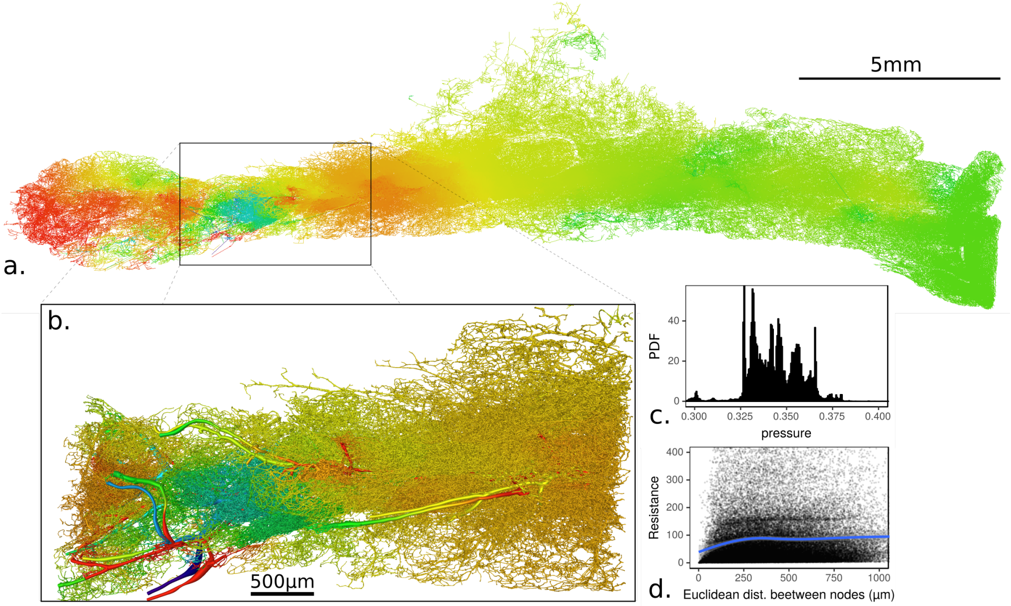

Fig A. Pressure distribution visualization. (**a**) Local pressure visualization from flow modeling into the vascular network. The pressure is color-coded with a cold-to-warm scale (from 0.3 to 0.4). (**b**) Zoom of main inlets/outlets localized in (a) and in Fig. 1a. (**c**) Distribution of pressures at network nodes. (**d**) Resistance versus Euclidean distance with mean displayed in blue and standard error in gray.
