# Supplementary material for: From whole-organ imaging to in-silico blood flow modeling: A new multi-scale network analysis for revisiting tissue functional anatomy
Source: PLoS Comput Biol. 2020 Feb 14;16(2):e1007322. doi: 10.1371/journal.pcbi.1007322 (PMC7062279; doi:10.1371/journal.pcbi.1007322)
Supplement: S2 Text — (PDF) [file pcbi.1007322.s002.pdf]

SI 2 Detailed results for all clustering weights

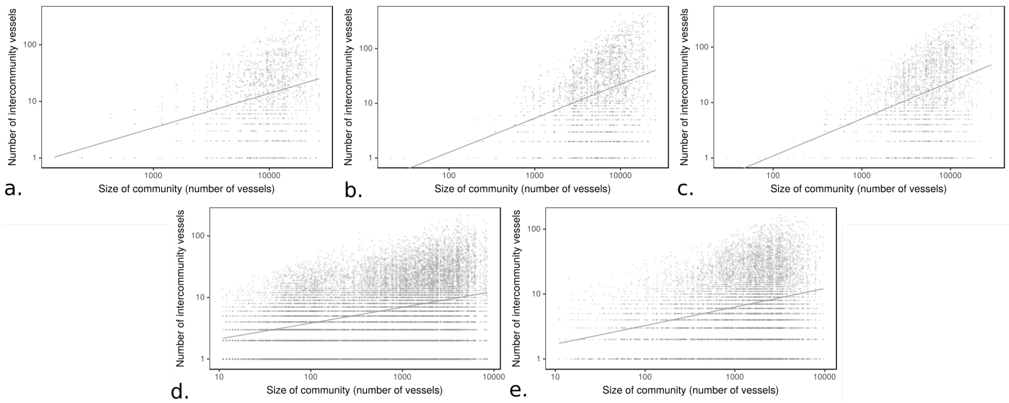

Fig B. Community strength comparisons without normalization. Gray filled area represents the strong community region between lines of slope 2/3 and 1. Dotted line is the linear regression of the scatter plot (gray points) of the number of vessels between each pair of communities versus the number of vessels in the community, for every clustering weight w_i: (**a**) w_0, no weight (slope s=0.61);(**b**) w_1, Euclidean distance (s=0.62); (**c**) w_2, geodesic distance (s=0.66); (**d**) w_3, hydraulic resistance (s=0.25); (**e**) w_4, hydraulic conductance (s=0.28).
