# Supplementary material for: From whole-organ imaging to in-silico blood flow modeling: A new multi-scale network analysis for revisiting tissue functional anatomy
Source: PLoS Comput Biol. 2020 Feb 14;16(2):e1007322. doi: 10.1371/journal.pcbi.1007322 (PMC7062279; doi:10.1371/journal.pcbi.1007322)
Supplement: S3 Text — (PDF) [file pcbi.1007322.s003.pdf]

SI 3 Community statistics

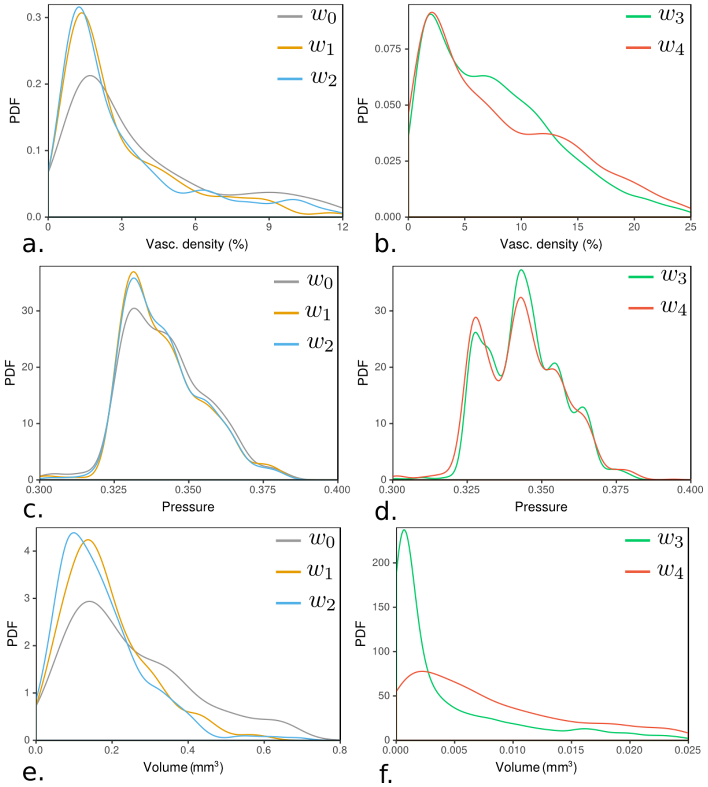

Fig C. Geometric and hydrodynamic statistics in vessel communities found by network clustering. (**a**) Vascular densities, (**b**) mean pressures and (**c**, **d**) volumes measured inside communities resulting from clustering of vascular graph $G_v(V_v, E_v, w_i)$ with w_i the edge weight function: w_0, -topological (unweighted) edges; w_1, -Euclidean distance separating vessel extremities; w_2 -geodesic distance separating vessel extremities; w_3, -vessel hydraulic resistance; w_4, -vessel hydraulic conductance.
