# Supplementary material for: From whole-organ imaging to in-silico blood flow modeling: A new multi-scale network analysis for revisiting tissue functional anatomy
Source: PLoS Comput Biol. 2020 Feb 14;16(2):e1007322. doi: 10.1371/journal.pcbi.1007322 (PMC7062279; doi:10.1371/journal.pcbi.1007322)
Supplement: S4 Text — (PDF) [file pcbi.1007322.s004.pdf]

SI 4 Further structural and functionnal analysis

We performed several additional investigations related to the graph clusters properties. Fig.D investigates two popular metrics used in graph analysis which were applied to the vascular network using structural weights w_2. This figure illustrates again that the structural weights offer the possibility to highlight the primacy of central region over peripheral ones. The investigated graph features are centrality and authority, both showing a more pronounced vascular connection, than the other part of the graph.

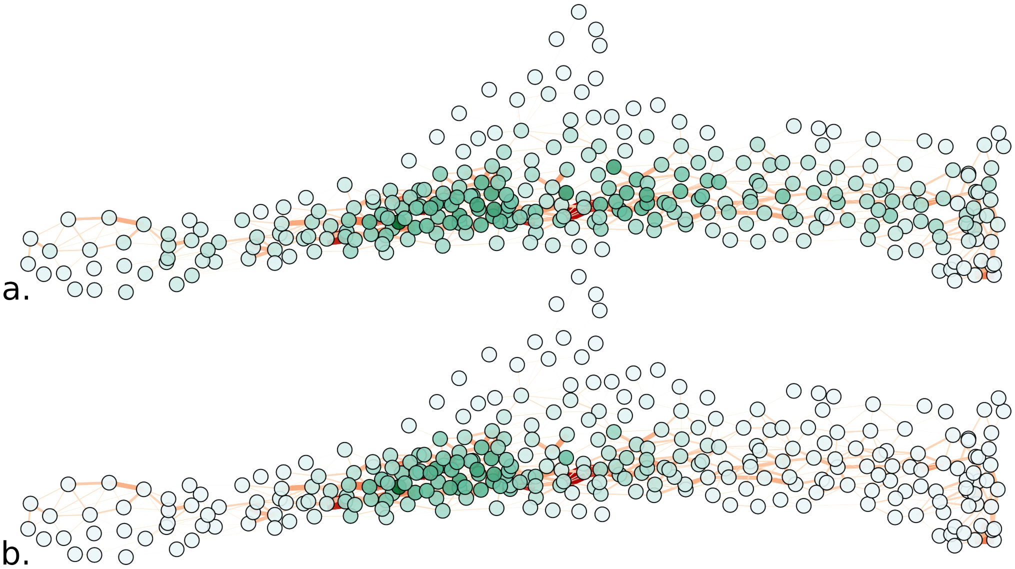

Fig D. Graph theory features extracted from the community adjacency graph $G_c^{w_2}$. The graph edges are sized according to the number of vessels connecting two adjacent neighbor communities and the nodes are colored (with a white-to-green scale) according to: (**a**) the centrality feature and (**b**) the authority feature.

Now considering other functional features of the same structural clusters, the vascular three hierarchy within each cluster is investigated in Fig.D. In Fig. E, we plot the topological distance to the main arterial/venous trunk, (i.e, the generation number). Furthermore, the larger the diameter, the larger the cluster. The core region exhibits small clusters with close relationship to the arterial domain. In the peripheral region, some clusters (not necessarily large ones) exhibit a hierarchy of vessels far from the main arterial trunk.

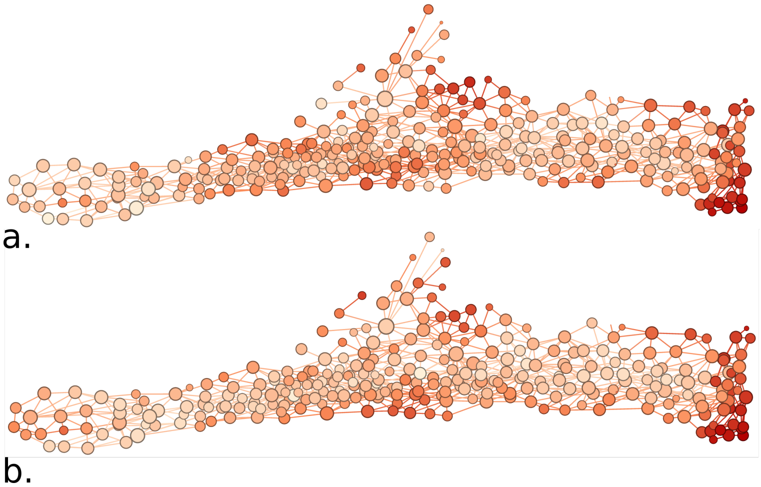

Fig E. Mean generation number per community starting from (**a**) arterial macro-vascular network and (**b**) venous macro-vascular network. Color map goes from light red to dark red for the lower to larger mean generation number inside each community with logarithmic scale. The diameter of each circle associated with each cluster is proportional to the cluster's volume.
