# Supplementary material for: From whole-organ imaging to in-silico blood flow modeling: A new multi-scale network analysis for revisiting tissue functional anatomy
Source: PLoS Comput Biol. 2020 Feb 14;16(2):e1007322. doi: 10.1371/journal.pcbi.1007322 (PMC7062279; doi:10.1371/journal.pcbi.1007322)
Supplement: S5 Text — (PDF) [file pcbi.1007322.s005.pdf]

SI 5 Details of flow modeling

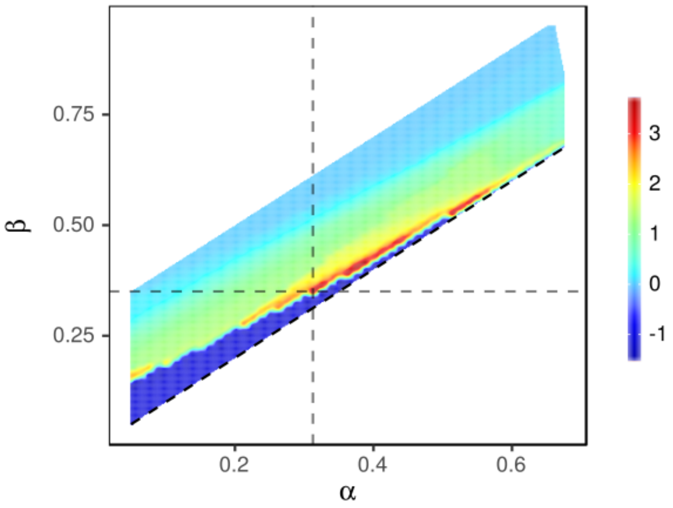

Fig F. Ratio of volumes perfused by the main inlet artery over all secondary inlet veins. The x-axis is the set of secondary outlet artery pressures, denoted α and the y-axis is the set of secondary inlet vein pressures, denoted β. Displayed volume ratios are interpolated for visualization purposes on a cold-to-warm scale. -1 represent parameters where the pair (α, β) results in one of the secondary inlets/outlets that did not follow the prescribed entering/leaving way. The set of parameters is such that $\alpha < \beta$ always resulted in this configuration (not displayed here). The bold dotted line is the separation line $\alpha = \beta$. The shaded dotted lines cross at the maximum volume ratio found: $(\alpha, \beta) = (0.35, 0.3125)$.
