# Supplementary material for: From whole-organ imaging to in-silico blood flow modeling: A new multi-scale network analysis for revisiting tissue functional anatomy
Source: PLoS Comput Biol. 2020 Feb 14;16(2):e1007322. doi: 10.1371/journal.pcbi.1007322 (PMC7062279; doi:10.1371/journal.pcbi.1007322)
Supplement: S6 Text — (PDF) [file pcbi.1007322.s006.pdf]

## SI 6 Kinetics computation

Although the perfusion problem is stationary one can compute the time taken to flow through a vessel by knowing the flow rate and the length of the vessel. The formula is written as

$$t_v^{loc} = \sum_{i=1}^{N_v^{el}} \frac{l_i S_i}{Q} \tag{1}$$

To obtain global times for each vessel, we then performed a breadth first search-like algorithm from every inlet (following the pressure gradient) and incremented the local time of the current vessel to the previous global time.

**Algorithm 1** Globalcomputation times

```
for every inlet do
    Tag all vertices as non-crossed
    Create queue
    Enqueue inlet vertex
    Compute global time of first edge
    Tag inlet vertex as crossed
    while queue is not empty do
        s = queue.dequeue()
        for every neighboor u of s do
            Compute global time on edge (s, u) : t^{glob}_{(s,u)}
            if u is a converging bifurcation then
                Get other vertex v that converges towards u
                Tag u as crossed
                if t^{glob}_{(v,u)} < t^{glob}_{(s,u)} then
                    Enqueue u
                else
                    Enqueue v
                end if
            else if u is a diverging bifurcation then
                Tag u as crossed
                Enqueue u

            end if
        end for
    end while
end for
```
