# Supplementary material for: From whole-organ imaging to in-silico blood flow modeling: A new multi-scale network analysis for revisiting tissue functional anatomy
Source: PLoS Comput Biol. 2020 Feb 14;16(2):e1007322. doi: 10.1371/journal.pcbi.1007322 (PMC7062279; doi:10.1371/journal.pcbi.1007322)
Supplement: S7 Text — (PDF) [file pcbi.1007322.s007.pdf]

SI 7 Effect of segmentation errors on perfusion exchanges between communities of vessels

To evaluate the impact of possible segmentation errors on the obtained results, we add Gaussian noise to the segmented diameter of a vectorized vascular graph. For each vessel, we add a random Gaussian variable (with zero mean, standard deviations of 0.5, 1, and 2) to the local vessel diameter corresponding to half, close to, and two times, respectively, the spatial resolution of the microscope. Two cases may appear non-realistically, thus transforming the network. The first case is when the added noise results in a segment element with a diameter < 0. In this case we keep the diameter with its original diameter. The second case concerns diameters becoming too small and possibly resulting in unrealistic hydraulic conductance. To deal with this issue, we set every segment element with a diameter $< 1\mu$m to exactly 1 (because it is the image resolution). The number of concerned segment element as a fraction of total segment elements numbers are reproduced in Table A for these two cases (columns 3 and 4).

Thus, we investigated the noise influence on the bi-functional community graph obtained from a geometrical weight clustering (w_2) because it is the most relevant one resulting from our study. We focused on two quantities: the number of resulting non-paired communities and the relative quadratic error on correctly paired error. Indeed, introducing noise can result in some communities no longer exchanging perfusion. Those communities were removed from the computation of the error, and their number is reproduced in Table A column 1. Relative quadratic error of flow for correctly paired communities is reproduced in column 3 of Table A.

For each noise level, the computations were run five times, and we summarized the results with the mean and the standard deviation of the data.

standard deviation	non paired communities	relative quadratic error	case 1 diameter	case 2 diameter
0.5	0.017± 1.73e-03	0.35±0.023	2.64e-05±3.97e-06	5.6e-04± 8.0e-06
1.0	0.031 ± 1.41e-03	0.38± 0.014	2.60e-04± 1.66e-05	1.6e-03± 5.53 e-05
2.0	0.044±3.3e-03	0.38 ± 0.027	7.72e-03±5.82e-05	0.014± 8.09e-05

Table A. Influence of Gaussian noise on diameters for perfusion of communities. Each row reproduces the mean and standard deviation of five different computations for different Gaussian noise levels on diameters.

When increasing the noise level up to two times the microscopic resolution, the error on perfusion exchange does not vary much, that is, up to 38%. However, the influence on the number of non-paired communities increases monotonously with the noise level, up to 4% of the total number of pairs of communities. These results indicate that the analysis done at the community scale in this work is quite robust to possible segmentation's errors done on diameters. Most communities still exchange blood with their neighboors and with a relative difference of 40%.
