# Supplementary material for: From whole-organ imaging to in-silico blood flow modeling: A new multi-scale network analysis for revisiting tissue functional anatomy
Source: PLoS Comput Biol. 2020 Feb 14;16(2):e1007322. doi: 10.1371/journal.pcbi.1007322 (PMC7062279; doi:10.1371/journal.pcbi.1007322)
Supplement: S8 Text — (PDF) [file pcbi.1007322.s008.pdf]

S8 Small adipocytes clusters versus functional clustering

Figure G illustrates the visual adipocytes structuration found in fat pads. To evaluate the biological relevance of communities revealed by our clustering, we investigated the spatial adipocytes distribution with respect to blood vessels within the mouse mice subcutaneous fat pad. This was doneWe used using double immunostaining of adipocytes (yellow cells, lipid droplet staining with Bodipy) and blood vessels (red, Lectin in vivo injection) as illustrated in Fig. G. Experiments were performed on 6- to 8-week-old male C57BL/6J mice (Harlan Laboratories) housed in a controlled environment. The mice were injected in vivo with retro-orbital rhodamine-red–labeled Griffonia (Bandeiraea) and Simplicifolia Lectin I (Eurobio Abcys) to achieve proper vessel labeling. At 30 min after lectin injection, animals were anesthetized by intraperitoneal injection of a ketamine/xylazine mix and then perfused intracardially with 4% para-formaldehyde solution. Fat pads were removed, post-fixed at 4°C overnight and kept in PBS at 4°C until cut into 300μm sections by using a vibratome (Campden). Sections were incubated with BODIPY™ 558/568 C12 (1:1000 in 0.2% triton X-100 in PBS, Invitrogen) at room temperature for 4 h. Imaging was using by confocal laser scanning microscopy (LSM510, Carl Zeiss).

From inspecting the illustrated sub-volume cut-outs (white dotted lines), one can observe convincing structural units, surrounded by vessels, in most-cases.

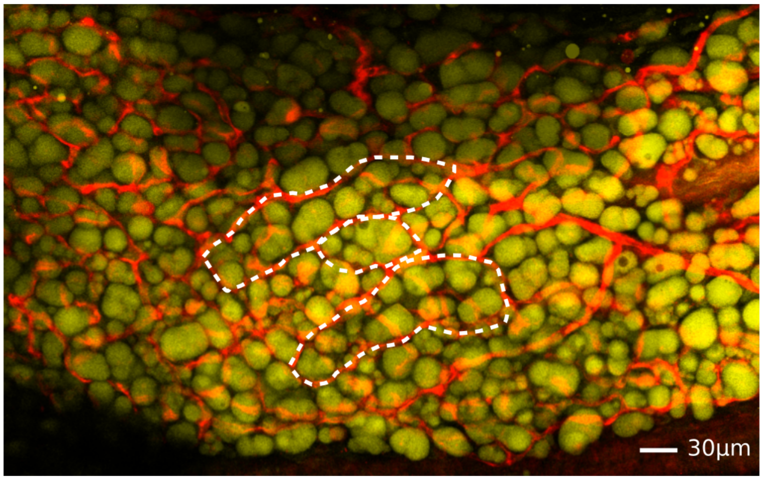

Fig G. Double labeling (blood vessels in red, adipocytes in yellow) fluorescent imaging of the mouse subcutaneous fat pad near a lymph node reveals small adipocyte clusters (three are exemplified by dotted white lines).

Furthermore, a precise quantitative study shows that the adipocytes of mice subcutaneous fat pad illustrated in Fig. G display a fairly narrow diameter fluctuations around the mean $d = 34\mu m \pm 9\mu m$ (diameter measured on 8868 adipocytes, $n = 5$ animals). Hence, the apparent large diameter variations of adipocytes in Fig.G are related to the apparent fusion of several adipocytes, due to convolution artefacts along the optical axis of the confocal imaging (a known caveat). A rough counting of adipocytes number in apparent clusters, qualitatively accounting for this fusion artefact, provide an average 13 ± 6 cells per clusters.
